# Food and drink advertising along school children’s transport routes in Victoria, Australia and policy implications

**DOI:** 10.1017/S1368980025000345

**Published:** 2025-04-10

**Authors:** Sherly X Li, Claire Hardi, Rebecca Godwin, Rachael Jinnette, Belinda Morley, Helen Dixon, Jane Martin

**Affiliations:** 1 Prevention Division, Cancer Council Victoria, Melbourne, VIC, Australia; 2 MRC Epidemiology Unit, University of Cambridge School of Clinical Medicine, Cambridge, UK; 3 Centre for Epidemiology and Biostatistics, Melbourne School of Population and Global Health, The University of Melbourne, Melbourne, VIC, Australia; 4 Centre for Behavioural Research in Cancer, Cancer Council Victoria, Melbourne, VIC, Australia; 5 Melbourne School of Population and Global Health, The University of Melbourne, Melbourne, VIC, Australia; 6 Melbourne School of Psychological Sciences, The University of Melbourne, Melbourne, VIC, Australia

**Keywords:** Children, Outdoor food and drink advertising, Food and drink marketing

## Abstract

**Objectives:**

To examine the extent and nature of food and non-alcoholic drink advertising displayed on public transport and infrastructure on school routes.

**Design:**

Audit of outdoor advertisements on government-controlled public transport and associated infrastructure (e.g. tram shelters, bus stops) on busy school routes in Victoria, Australia. Using a strict protocol, trained field workers collected data on the type and content of outdoor advertising during February 2023 (start of school year). Food/drink advertising was classified (unhealthy or healthy) according to the Council of Australian Governments Health Council National interim guide to reduce children’s exposure to unhealthy food and drink promotion (2018).

**Setting:**

Government-controlled buses, trams and public transport infrastructure on routes from eleven of the busiest train stations in metropolitan Melbourne and regional Victoria, Australia to fifty public primary and secondary schools. Stations were chosen based on annual patronage, area-based socio-economic area (SEA) and regionality).

**Results:**

156 out of 888 advertisements were for food and non-alcoholic drinks. Of these, almost six in ten (58 %) were deemed unhealthy irrespective of SEA or regionality. Marketing appeals most featured were taste (31 %), convenience (28 %) and emotion (9 %). A significantly higher proportion of unhealthy advertisements were displayed within 500 m of schools *v*. outside this radius (91 % *v*. 57 %, *P* < 0·01).

**Conclusion:**

Given the detrimental impacts of exposure to unhealthy food/drink advertising on children’s diets, the pervasive, powerful presence of such advertising across government public transport assets, particularly around schools, contradicts public health recommendations to protect children from exposure to and influence by this harmful marketing and warrants government action.

Strong and consistent evidence demonstrates that children and adolescents are vulnerable to the effects of food marketing^(1)^ which increases their preferences for, and intake of, marketed foods^(2)^. Despite recommendations by the World Health Organization for governments to regulate to protect children from unhealthy food and drink marketing^(3)^, an international review of outdoor food marketing, including on public transport used by children to travel to school, found that the majority of food advertisements were for unhealthy products^(4)^.

The WHO highlights two factors that underpin effective marketing^(3)^. Firstly, marketing exposure is based on reach (percentage in a target market exposed over a specified period) and frequency of an advertisement (how many times the average person is exposed). Secondly, power refers to the extent marketing achieves its communication objectives, measured by creative content/strategy (e.g. emotional appeals, promotional characters), which affects children’s food choices and intake^(2)^. Outdoor advertising in popular settings can achieve high reach and frequency of exposure, employing artful persuasive appeals to build brand awareness and nudge consumers along the path to purchase.

Currently in Australia, only the Australian Capital Territory has adopted a mandatory policy to restrict unhealthy food and drink advertising on bus and light rail networks (2016)^(5)^. Audits of outdoor food/drink advertising around schools and on bus and train networks in other Australian jurisdictions reveal a concerning proportion of unhealthy food/drink advertising (84 % within the Sydney train network and 70–80 % around Perth schools)^(6–8)^. One study examined the prevalence of food advertisements in metropolitan Melbourne, Victoria, surveying 588 public transport stops within 10 suburbs, conducted in 2013^(9)^. Of 233 food/drink advertisements identified, they noted socio-economic patterning in the nature of the advertisements, showing that advertisements for unhealthy fast food chains, fruit juice and flavoured milk were more common in most disadvantaged areas^(9)^.

However, there is no recent data from metropolitan (since 2013) and no surveys of regional Victoria. We aimed to: i) quantify the extent and nature of food and drink advertising displayed on government-controlled public transport and infrastructure on selected routes to schools, ii) determine if there is a socio-economic and regional difference in the extent of unhealthy marketing and iii) characterise the potential impact of this marketing, according to WHO, by examining potential exposure to product, brand, healthiness and power of the advertising (persuasive marketing appeals).

## Methods

An audit of outdoor advertisements on government-controlled public transport vehicles and infrastructure on routes to schools in eleven locations across Victoria, Australia. Our focus was on public transport routes to schools (not schools), and we included only outdoor advertising (format). To ensure policy relevance to government-controlled assets, we excluded advertisements on commercial property e.g. vending machines, telephone booths and retail stores at bus/tram stops and train stations. To ensure consistency in the collection and classification of advertisements, data were collected following strict, standardised study protocol (see online supplementary material, Supplementary Material 1) by trained field workers (detailed in see online supplementary material, Supplementary Material 2). An objective criterion was used to classify the advertisements and where there was ambiguity, a decision was made upon consensus with senior authors who checked all data.

### Sampling

Purposive sampling ensured coverage of contrasting areas by regionality and socio-economic area (SEA) as defined by relative socio-economic disadvantage, rather than representation of Victoria. Locations were chosen based on the busiest train stations according to annual patronage data (Department of Transport and Planning), when ranked within the two lowest (quintile 1 and 2) and one highest (quintile 5) SEIFA quintiles, respectively (denoting SEA),^(10,11)^ and by metropolitan and regional Victoria. Of the eleven locations, seven were metropolitan and four regional. Six were classified as being within low SEA (Bendigo, Melton, Springvale, Sunshine, Dandenong, Clayton), one medium (Watergardens) and four within high SEA (Macedon/Gisborne, Geelong, Essendon, Flinders Street area in Melbourne). Fifty primary and secondary schools in these locations were selected, based on the highest density according to student enrolment data (Department of Education and Training)^(12)^ and in the closest proximity to the train station. Three percent of schools in Victoria were represented in this audit. Tram and bus routes with the shortest or near shortest distance from the train station to the school were identified using Google Maps.

### Audit tool and data collection

A digital data collection tool, based on previously validated INFORMAS protocol^(13)^, was developed for data collection via smartphones (see online supplementary material, Supplementary Material 1). For each advertisement, a photo was uploaded, and the characteristics of the advertisement were recorded. Some characteristics of the advertisement were collected in the field and others were inferred from the photo and location (distinction of which is found in see online supplementary material, Supplementary Material 3).

Data collection occurred during the school term in February 2023. All advertisements visible in public areas and intended to sell goods/services were captured and included banners, hoardings, signs, images or rolling static displays, digital billboards and panels with video images, movable billboards and displays^(14)^. Advertisements were collected for each of the eleven locations at train stations and at bus and tram stops along the most direct transport route to a school. Additionally, advertisements were captured on the outside of moving buses and trams within a 90-min window at the bus/tram stop closest to the defined (delete this word) schools of interest during peak times (8–9·30 am or 2·30–4 pm weekdays) when students travel to or from school to reflect their potential advertising exposure. Potential exposure is defined as the maximum possible exposure based on all advertisements displayed at a location.

### Data classification

Food and non-alcoholic drinks advertisements were classified according to the Council of Australian Governments (COAG) Health Council national interim guide for food promotion (2018) (see online supplementary material, Supplementary Material 4)^(15)^ supplemented by INFORMAS^(13)^. Advertisements were classified as i) unhealthy: ‘foods not recommended for promotion by the COAG’, covering seven sub-categories: sugar-sweetened drinks and artificially sweetened drinks; flavoured milk; confectionary; savoury snacks; sweet snacks; ice-creams/desserts and unhealthy meals. ii) healthy: foods that did not fall under the above category and iii) not applicable: those that fell outside of the COAG guidelines including specialised foods (e.g. baby foods) or fall outside of the scope of Australian Dietary Guidelines classification (e.g. stock cubes, herbs, dietary supplements, tea/coffee). For food/drink advertisements without a food/drink image (we termed this ‘brand only advertisements’), the brands were classified as unhealthy if the foods/drinks they sold were predominantly classified by COAG as unhealthy e.g. KFC and Coca-Cola. Each advertisement was examined for creative content (a measure of power) (see online supplementary material, Supplementary Material 3), where multiple measures could apply to one advertisement.

### Data analysis

Descriptive analysis examined the number, location, type, size and power (creative content and promotional strategies) of the advertisement. Differences in the proportion of healthy and unhealthy advertisements were examined using Fisher’s exact tests according to location (metropolitan *v*. regional), SEA (low *v*. medium/high), size (small: ≥A4 by <1·3 × 1·9 m, *v*. medium: >1·3 × 1·9 m but <2·0 × 2·5 m, *v*. large: >2·0 × 2·5 m) and distance from a school (within *v*. outside of 500 m radius). Analysis was performed in Stata/MP 16·1.

## Results

In total, 888 advertisements were recorded from eleven train stations, 279 bus and tram stops and 245 moving buses and trams on school routes across Victoria in February 2023. Of these, 156 were for food and non-alcoholic drinks, with the majority deemed unhealthy (58 %, *n* 90 of which 3 were brand only) and a minority deemed healthy (35 %, *n* 55) or not applicable (7 %, *n* 11). The top three product categories advertised were unhealthy meals e.g. KFC-Cola BBQ Wings (32 %, *n* 49), followed by vegetables (18 %, *n* 28) and sugar-sweetened drinks (15 %, *n* 24).

Nearly half (44 %, *n* 40) of all unhealthy food/drink advertisements were located at tram stops, almost one-third (29 %, *n* 26) on buses, followed by train stations (16 %, *n* 14) and bus shelters (11 %, *n* 10). Flinders Street area (City of Melbourne) recorded the most food/drink advertisements (*n* 99) and displayed just over half (51 %, *n* 46) of all unhealthy food/drink advertisements across the audit.

Table 1 shows whether the proportion of unhealthy food/drink advertising differed by regionality, SEA, distance from school and size of the advertisement. There was a significantly higher proportion of unhealthy advertisements within 500 m of schools (91 %) compared with those outside of the 500 m radius (57 %, *P =*0·002).

**Table 1.** Healthy compared with unhealthy food and non-alcoholic drink advertisements by domains assessed

Domain	Unhealthy advertisements (*n* 90)		Healthy advertisements (*n* 55)		*P*-values^*^
	*n*	%	*n*	%	
Regionality					
Metro Melbourne	86	*60·99*	55	*39·01*	0·298
Regional Victoria	4	*100·00*	0	*0·0*	
Socio-economic area					
Medium/high	29	69·05	13	30·95	0·346
Low	61	59·22	42	40·78	
Distance from school^†^					
Within 500 m	21	*91·3*	2	*8·7*	0·002
Outside 500 m	69	*56·56*	53	*43·44*	
Size of advertisement^‡^					
Small/medium	64	*62·75*	38	*37·25*	0·852
Large	26	*60·47*	17	*39·53*	

* Fisher’s exact test for difference in proportions.

† Distance from school (in km) was estimated using tram and bus routes with the shortest or near shortest distance from the train station to the school via Google Maps.

‡ Size of the advertisement was estimated visually, comparing to examples of small *v*. medium *v*. large.

Sample size: *n* 145 (healthy = 55; unhealthy = 90 advertisements). Of 156 advertisements, eleven had missing Council of Australian Governments (COAG) classifications so were dropped from the analysis.

Regarding the frequency of brands, Uber Eats was the most represented brand within the audit (26 %, *n* 40 of all food/drink advertisements) followed by Health and Wellbeing Queensland (15 %, *n* 24) and 7-Eleven (13 %, *n* 20). Uber Eats had the highest frequency of brand exposure out of all food/drink advertisements across the audit (26 %), the subgroup of unhealthy advertisements (40 %) and unhealthy advertisements within the Flinders Street area (78 %). The highest number of Uber Eats advertisements was recorded on one single route from a major train station to a primary school: thirty-one advertisements (see online supplementary material, Supplementary Fig. 1).

A total of 148 marketing strategies were featured across ninety unhealthy food/drink advertisements. The main creative strategies employed were appeals to the following: taste (31 %, *n* 46), convenience (28 %, *n* 42) and emotion (9 %, *n* 13) (see online supplementary material, Supplementary Fig. 2).

## Discussion

This audit examined the extent and nature of unhealthy food and non-alcoholic drink advertising on government-controlled public transport and assets that children are potentially exposed to as they travel to and from school within Victoria. The majority (58 %) of food/drink advertisements featured unhealthy products and of these, nearly half (47 %) promoted unhealthy meals or sugary drinks. This undermines public health recommendations in promoting a healthy diet and underscores the importance of protecting children from the power and influence of food and drink marketing that contributes to unhealthy diets.

Similar surveys conducted around Australia found between 74 and 87 % of food advertisements were for unhealthy products, somewhat higher than our finding (58 %)^(7,8,16,17)^. It is possible that our audit yielded a lower proportion because the Outdoor Media Association and Health and Wellbeing Queensland ran a vegetable campaign at the time which may have inflated the healthy category. The other possibility is that while not explicitly stated, these other studies may have included advertising on non-government assets (e.g. vending machines) resulting in a higher proportion of unhealthy advertising. There is still considerable room for reorienting the food marketing landscape towards promoting healthy over unhealthy food and drink products, which currently dominate.

While an earlier audit in Victoria found some unhealthy food products were more commonly advertised in disadvantaged suburbs (e.g. fast food chains, flavoured milk and juices)^(9)^, we found no significant difference in the proportion of unhealthy food advertisements by SEA. However, the survey found a significantly higher proportion of unhealthy advertisements within a 500 m radius of schools compared with those outside this (91 % *v*. 57 %). Similarly, a WA study found that 70 % of schools surveyed had unhealthy food/drink advertisements within 500 m^(6)^, and a study by Kelly *et al.* noted twice the density of unhealthy food/drink advertisements closer to schools in NSW^(18)^. Internationally, unhealthy food marketing is also more prevalent closer to schools^(19)^. Further, within this audit, the highest density of food advertisements was recorded in central Melbourne’s Flinders Street area (63 % of all food/drink advertisements, *n* 99) with unhealthy food advertisements representing more than half (51 %, *n* 46) of unhealthy advertisements across the entire audit. This demonstrates the intentional placement of unhealthy food advertising in transport hubs with higher commuter throughput (Flinders Street Station has the highest annual patronage)^(10)^. Together these findings indicate that there is strategic placement of unhealthy food advertising where it is more likely to achieve high reach and frequency of exposure, by children/teenagers near schools and in high throughput transport hubs.

The WHO recommends that the goal of policies to protect children from food marketing should be to reduce the exposure and power of unhealthy food marketing^(3)^. In our audit, Uber Eats was the most prominent unhealthy food advertiser (26 %) and had thirty-one advertisements along a single school route. This highlights the strategic placement of advertisements on children’s school routes, with high exposure linked to strong brand recognition and consumer loyalty. Food companies deliberately target children, recognising them as potential lifelong, loyal customers with emerging evidence showing adolescents may be especially responsive to junk food marketing appeals^(20)^. Australian adolescents who purchase food/drink on their school commute or outside school grounds during school hours are known to purchase more unhealthy discretionary foods generally^(21)^, and the present audit suggests the food advertising environment on school routes may contribute to this phenomenon. Moreover, greater exposure to food delivery platforms may instil a preference for convenience and takeaway over making home-cooked meals. These together pose concerning implications on children’s intake and subsequently contribute to diet-associated chronic diseases. Few Australian studies have examined marketing strategies used in outdoor advertising. In this audit, the creative strategies most used to advertise food/drinks appealed to taste, convenience and emotion, which have previously been identified as having persuasive power with children^(3)^. This finding is consistent with a recent review on outdoor food marketing^(4)^ and highlights how marketing applies known determinants of food choice to influence purchase and intake^(22)^. Our findings echo international reports^(19)^.

The strengths of our audit include using previously validated protocols, training of data collectors and the use of a standardised protocol, as well as being the first exploratory examination across both regional and metropolitan locations in Australia. Limitations include purposive sampling, confining the generalisability of our findings. Further, the number of advertisements sampled may have limited the statistical analyses. Despite this, we note novel findings that provide direction for future research and advocacy.

### Policy considerations

This analysis excluded advertisements on commercial property on or next to train stations and bus/tram shelters (*n* 114 on vending machines, telephone booths and retail stores), most of which were for unhealthy food (97 %, data not shown) – an even higher proportion than on government-controlled property (58 %). Given their contribution to potential exposure, unhealthy food and drink advertisements on commercial as well as government-controlled property should be considered if policies to restrict marketing within public transport settings are to have the largest impact. Also, studies have found that when brands who predominantly sell unhealthy food/drinks advertise healthy options or display branded advertising alone, this can still prompt consumers to increase their intake of unhealthy food/drink^(23,24)^ and may be used by companies to circumvent policies to restrict unhealthy food/drink advertising. Therefore, policies should have separate recommendations for classifying and regulating ‘brand only’ advertisements. A recent international review found mandatory policies are more likely than voluntary policies to reduce both exposure and the power of food marketing^(25)^. Mandatory policies for outdoor advertising such as that for Transport of London provide important precedents and are recommended by the WHO^(3,26)^. The London ban led to an average 1000 calorie decrease from weekly unhealthy food and drink purchases compared with no ban^(26)^, with no negative impacts on advertising revenue^(27)^. Similar policies have been implemented in many councils across the UK. This supports the case for implementation in Australia. We therefore recommend a mandatory policy banning unhealthy food/drink advertisements within 500 m of schools and on public transport and associated infrastructure in Australia, with compliance monitored and enforced.

Our findings reveal that Victorian children are potentially exposed to high levels of unhealthy food/drink advertising as they travel to and from school. Addressing this by removing unhealthy food marketing is an important step in protecting our children from the power and influence of this harmful, predatory marketing and enabling a cultural shift toward healthier food preferences for lifelong health.

## Supporting information

Li et al. supplementary material Li et al. supplementary material

## Supplementary material

For supplementary material accompanying this paper visit https://doi.org/10.1017/S1368980025000345
