# Supplementary material for: Food and drink advertising along school children’s transport routes in Victoria, Australia and policy implications
Source: Public Health Nutr. 2025 Apr 10;28(1):e97. doi: 10.1017/S1368980025000345 (PMC12171914; doi:10.1017/S1368980025000345)
Supplement: Li et al. supplementary material [file S1368980025000345sup001.docx]

**Supplementary material 1: Food Fight study protocol for data collection and coding**

**Key research questions**

Total number of advertisements recorded

Total number of food/drink advertisements recorded and proportion to total advertisements

From the food and non-alcoholic drink advertisements

- Number and proportion of advertisements that are healthy vs unhealthy
- Number and proportion of advertisements by types of foods/drinks
- Number and proportion of types of brands within brand only advertisements
- Number and proportion of healthy foods advertised by unhealthy brands
- If the number (proportion) and mean (SD) number of healthy vs unhealthy advertisements differ by the following factors
  - - Size of advertisement
    - Location: metro vs regional Victoria, SES/inequity- based on SEIFA
    - Distance from school
- Exposure: frequency of advertisement of the same product in a specific location
- Power: mean number of healthy vs unhealthy advertisements with marketing creative

**Methods**

**Location/sampling:**

- Purposive sampling:
  - - Classify all train stations by SEIFA quintiles (using only the highest -5^th^ quintile and lowest SEA categories- first 2 quintiles) and metro vs regional Victoria. Choose the busiest train stations according to SEIFA and metro vs regional Victoria.
    - Busiest train station selection will be based on how many people frequented according to data from [Department of Transport on Annual Metro/Regional station patronage](https://discover.data.vic.gov.au/organization/department-transport-planning). Date of data used: annual data for 2020-21
  - Definition of busiest: based on annual number of passengers according to myki ticketing data
    - In total we would have 11 train stations, representing around 7 metro, 4 regional and 4 from the high, 1 from medium and 6 from the low SEIFA categories.
    - Identify a public primary and a secondary school with the highest number of students within 3km radius of each train station (according to enrolment data by the [Department of Education](https://discover.data.vic.gov.au/dataset/all-schools-fte-enrolments-feb-2022-victoria)).
    - Identify all bus and tram stops between the station and school
    - Where a location does not have a mode of transport, more schools will be identified so a mode of transport may be oversampled (e.g. Bendigo doesn’t have trams, so 4 bus routes are sampled)
  - In total, we will survey 11 train stations, each covering at least 4 routes between a train station and a school (either by bus, tram, or both) with a total of 50 routes.
- An example is provided below

**Example of route selection for Bendigo**

Station: Bendigo

Address: Railway Pl, Bendigo VIC 3550

SEIFA: IRSAD 2- is classified as lowest SEA (postcode 3550) ([ABS look up](https://www.abs.gov.au/ausstats/abs@.nsf/Lookup/by%20Subject/2033.0.55.001~2016~Main%20Features~IRSAD%20Interactive%20Map~16))

Metro/Regional: regional

We will survey 4 routes per train station (1 primary school: 1 tram, 1 bus and 1 secondary school: 1 tram and 1 bus route). If a location does not have a certain mode of transport, extra schools will be added to make up to 4 routes.

| **School** | **Address** | **Student number** | **Kms from school (driving)** | **Bus route from train station to school** | **Tram route from train station to school** |
| --- | --- | --- | --- | --- | --- |
| **Primary** |  |  |  |  |  |
| Spring Gully Primary School | 104-110 Spring Gully Road | 412 | 3.3km | 65 | NA |
| Camp Hill Primary School | 50 Gaol Rd | 295 | 1.6km | 52/ 53/ 55 | NA |
| **Secondary** |  |  |  |  |  |
| Bendigo Senior Secondary | 40 Gaol Road | 1727 | 2.4km | 52/ 53/ 55 | NA |
| Bendigo South East 7-10 Secondary College | 56 Ellis Street | 1321 | 2.7km | 62/ 65/ 70 | NA |

**Settings eligible:**

- Outdoor marketing on government owned public transport or public transport infrastructure
  - public transport vehicles (bus, tram): observation within a 30minute window between peak hour times for student travel and reflective of what students may be exposed to on their journeys to school (8.00-9.30am or 2.30-4.00pm, Monday – Friday, according to [VicRoads](https://www.vicroads.vic.gov.au/safety-and-road-rules/road-rules/a-to-z-of-road-rules/school-speed-zones)) at the closest stop to the school for that school route
  - public transport infrastructure e.g. bus and tram stops where a shelter is present, the train station concourse, station platform, on the floor, cross-track billboards, or any external advertisements designed to be seen by commuters standing on the platform or entering/exiting the station.
- Exclusion:
  - where a location e.g. bus stop with a pole but does not include any infrastructure to advertise.
  - advertisement on the exterior surfaces of commercial property on public transport infrastructure e.g. vending machines, telephone booths, retail shop or restaurant on the concourse of a train station.

**Eligible advertisement:**

- All advertisements which is a sign that is visible in public areas, with branded information and intended to sell goods/services. It can be food or non-food.
- Advertisement formats include stationary signs/objects that are print, digital, video, and on exterior surfaces of buses and trams.
- Examples: banners, hoardings, signs, images or rolling static displays, digital billboards and panels with moving or video images, movable billboards and displays, as per [alcohol advertising regulations near schools](https://www.vgccc.vic.gov.au/liquor/pub/understand-your-liquor-licence/responsible-alcohol-advertising-and-promotion). It also may include temporary flyers and branded furniture.
- It includes displays promoting a product, brand/logo or shop e.g. Coca Cola logo. It is important to capture brands even if they do not depict specific foods.
- To capture all advertisements because advertisement not directly targeting children could still appeal to children.
- Definition of food and drink advertisement: all food and non-alcoholic beverage products or companies
- How to count
  - Rotating billboards: all advertisements shown within one complete rotation should be recorded.
  - Vending machines: count each advertisement separately e.g. if same advertisement occurs on both sides, count twice.
  - Multiple billboards with a connected image, count as 1
- Exclusion:
  - Historic ads preserved as artwork, unbranded signs
  - Advertisements that are unbranded or symbols that are mainly used for store identification e.g. McDonald’s arches outside a McDonald’s store.

**Data collection (in the field)**

- Data collectors will walk the specified routes and visually scan for advertisements at eligible settings, then complete the digital data collection tool for each advertisement
- There were at least 2 data collectors per location/route, to ensure safety and helping to verify data collected.
- Each data collector is given the following:
  - Access to the digital data collection tool to use on their smartphones
  - Map of train stations and all routes to local schools that were designated to them
  - Document list of each bus stop name on the route that were designated to them
- Digital data collection tool:
  - accessed via a link on their phone to collect data via a platform on the Cancer Council Victoria website.
  - Data collected will be exported into Excel.
  - Tool will be based on previously validated tools according to the [INFORMAS protocol](https://cancervic.sharepoint.com/:b:/r/sites/Prevention-ObesityCampaign/Shared%20Documents/General/2023%20Campaign/Research%20and%20Evaluation/Publications/INFORMAS_Outdooradvertisingprotocol_2017.pdf?csf=1&web=1&e=6lKK2d).
  - Tool website: [https://www.cancervic.org.au/foodfight/take-action/data-collection](https://url.au.m.mimecastprotect.com/s/AHFZCYW8WOTjAqLqi0fAHxsGuv?domain=cancervic.org.au)
  - Containing elements collected in the field, including capturing a photo of the advertisement
- If an eligible location has any infrastructure to advertise, even if it does not have any food or drink advertisement, this must be noted. Do not record for locations without any infrastructure to advertise e.g. tram/bus stops without a shelter where advertisements can be posted.
- Teams chat platform is set up to allow data collectors to post questions and resolve them with senior researchers. All decisions will be logged and visible for all other data collectors who may come across similar issues.

**Data cleaning and coding (in the office)**

- data will be exported into Excel
- each data collector must review and clean their data where necessary on the day of data collection to ensure accuracy
- using the photo of the advertisement, data collectors are to complete data coding for the rest of the data collection elements that can be inferred from the photo (see Supplementary Material 3)
- a standardised coding template was used, with a limited set of options to minimise inconsistencies due to ambiguity
- any queries are to be raised on the day with senior researcher and decisions posted onto
- summary of decisions and any other questions to be discussed at bi-weekly project team meetings referring to photos

**Food/drink classification:**

- Each food/drink advertisement will be classified into two overarching categories according to COAG Health Council National Interim guide to reduce children’s exposure to unhealthy food (Supplementary Material 4):
  - Unhealthy foods: are ‘Food not recommended for promotion by the COAG’ according to the Australian Dietary Guidelines and the Australian Guide to Healthy Eating. They include seven sub-categories: sugar sweetened drinks, flavoured milk, confectionary, savoury snacks, sweet snacks, ice-creams/desserts, and unhealthy meals.
  - Healthy foods: those that do not fall under the first category
  - Additional items: although the COAG definition did not specifically include flavoured milk as an example under sugar-sweetened drinks, it was included as a separate category as it complies with the definition of sugar-sweetened drinks provided in the COAG interim guide.
- Brands without direct reference to a food/drink and stores will be classified as unhealthy if the foods/drinks they sold were predominantly classified by COAG as unhealthy e.g., KFC and Coca-Cola and/or to expert consensus.
- Alcohol will not be included as a food/drink because of the audience age group of interest.

**Supplementary material 2: Food Fight data collector training program**

This is a high level summary of the standardised training program (3 x 2hr sessions) provided to all data collectors. Additionally, we had regular meetings to discuss and resolve queries.

**Pre data collection training**

Two 2-hour training was provided with in-depth PowerPoint slides. A summary document of the training was also provided at the end.

1. *Project Tasks and Allocations*

- Outline of field project tasks including the group sizes and main tasks.
- Equipment needed; smart phone, walking shoes, sunscreen, food, water, project related documents.
- Allocations of train and tram stations and bus routes.
- **Resource provided:** Maps of train station allocation that also including bus routes to the local schools.
- **Resource provided:** Document list of the bus stop name on each route that highlighted which bus stop the 30-minute observation window would occur.

1. *Settings definition*
   - **Key Learning:** What is public transport infrastructure and what is excluded.
   - **Practical:** Examples of advertisements at trains stations
2. *Advertisement definition*
   - **Key Learning:** Inclusions and exclusions of types of advertisements
   - **Practical:** How to define advertisements with examples pictures at train stations
   - How to collect data for digital rotating billboards and vending machines.
3. *Data Collection*
   - **Key Learning:** How to use the data collection tool.
   - **Key Learning:** What is a food / drink advertisement.
   - Summarise the data to be collected for a food or non-food advertisement.
   - **Key Learning:** How, where and when to conduct the 30-minute observation.
   - **Resource provided:** Bus audit data collection template with example.
4. *Additional Information*
   - Outline of which bus stop are used for the 30-minute observation audit.
   - Suggestions on how to plan the routes.
   - **Resource provided:** Audit protocol
   - **Practical:** 1-hour demonstration of using the data collection tool at a local tram stop with a senior researcher. Data collected were checked for accuracy and queries were discussed.

**Post data collection training 1 – Data coding**

Two 2-hour training was provided with in-depth PowerPoint slides. A summary document of the training was also provided at the end.

1. *Data cleaning*
   - Outline of the office project tasks.
   - Data cleaning tasks step by step.
   - **Key Learning:** How to clean the data using excel.
2. *Data coding*
   - How to enter each variable for the food and drink advertisements with example advertisements to demonstrate this.
   - **Resource provided:** Data coding template with each code and variable.
   - **Resource provided:** Food Categories spreadsheet outlining what food categories were considered non-healthy and healthy with examples.
   - Summary of the codes.
   - How to use the decision log when changes were made, or difficult questions resolved.
   - **Resource provided:** Decision log spreadsheet with an example.
3. *Data classifications*
   - Each variable and an example of how-to code for a specific advertisement.
   - **Resource provided:** A master spreadsheet for brand store names to be used for consistency.
   - **Practical**: Work through examples of marketing types.
   - How to calculate distance from school.
   - **Practical:** Practice how to code each advertisement using the code

**Post data collection training 2 – Data Analysis of simple analyses e.g. proportions (more complex analyses undertaken by senior researchers)**

A 2-hour training was provided with in depth PowerPoint slides.

1. *Data analysis*
   - Provided reference/reading materials of previous research conducted on this topic.
   - Provided our research questions.
   - Ways to present data in tables and graphs
   - **Practical:** Demonstration on how to conduct the analysis in excel, including what functions to use.
   - **Practical:** 20-minute daily session to discuss any results

**Supplementary material 3: Food Fight Data Collection Elements**

**Collected in the field: within the digital audit tool**

| **Characteristics** | **Example/ options** |
| --- | --- |
| Add ID | 325027 |
| Data Collectors Name | John |
| Date | 16/02/2023 |
| Time | 11:15:00 AM |
| Location/Station/Stop Name | Flinders Street Station |
| School this transport route is on | N/A if at a station  Free text if at a bus/tram stop |
| Metro vs regional | Metro |
| If at a train station, number of platforms | 13 |
| Is it Food or Drink* | Yes / No |
| Size of Advertisement | Small (>A4 but <1.3m x 1.9m)  Medium (>1.3m x 1.9m but <2.0m x 2.5m)  Large (> 2m x 2.5m) |
| Setting | On a bus/ On a tram / On a train / Tram stop / Train Station / Attached to a non-food shop or business / Near food shop / Near another venue used by children / Free Text option |
| If at a train station, where was the advertisement located | Concourse / Station platform / On the ground / Cross-track billboard / External to the station / Free Text |
| Advertisement format | Billboard / Free-standing / Painted building or wall / Digital / Merchandising / Vending machine / Other |
| Is it an alcoholic drink | Yes / No |
| Is it brand only (no direct image of food or drink) | Yes / No |

**Collected during analysis: inferred from the advertisement photo or location**

| Characteristics | Example / options |
| --- | --- |
| Food or drink category | Sugar Sweetened Beverage / Diet Soft Drink / Confectionery / Savoury Snacks / Sweet Snacks / Desserts, ice creams, ice confections / Unhealthy Meals / Wholegrains / Fruit / Vegetables / Dairy Products / Meat and Alternatives / Oil / Water / Baby Food / Alcohol / Recipe Additions / Dietary Supplements / Processed meats / Healthy meal / Tea and coffee / NA |
| Product Name | Coca-Cola |
| Brand store name | Vending machine with coke bottle on it saying “next stop ice cold refreshment” |
| Marketing type | Promotional characters or celebrity / premium offers / competition / Appeals to price / Appeals to taste / Appeals to emotion / Reference to sport / Reference to local culture / Reference to health / New / modern / Appeal to convenience / Appeal to image / Social corporate responsibility / Other / None |
| Is it classified as healthy or unhealthy by COAG? | No / Yes / NA / Alcohol |
| Healthy Food Unhealthy Brand | Yes / No |
| Incidental Unhealthy Food (food on an advertisement for a non-food brand) | Yes / No |
| Distance from school | 2000m |
| Is it within 500m of a school? | Yes / No |
| Is it within 1000m of a school? | Yes / No |

*For non-food advertisements, data was entered until the question ‘Is it Food or Drink’, whereas for food/drink advertisements all the above data were captured.

**Supplementary material 4: The Council of Australian Governments (COAG) Health Council national interim guide for food promotion (2018)**


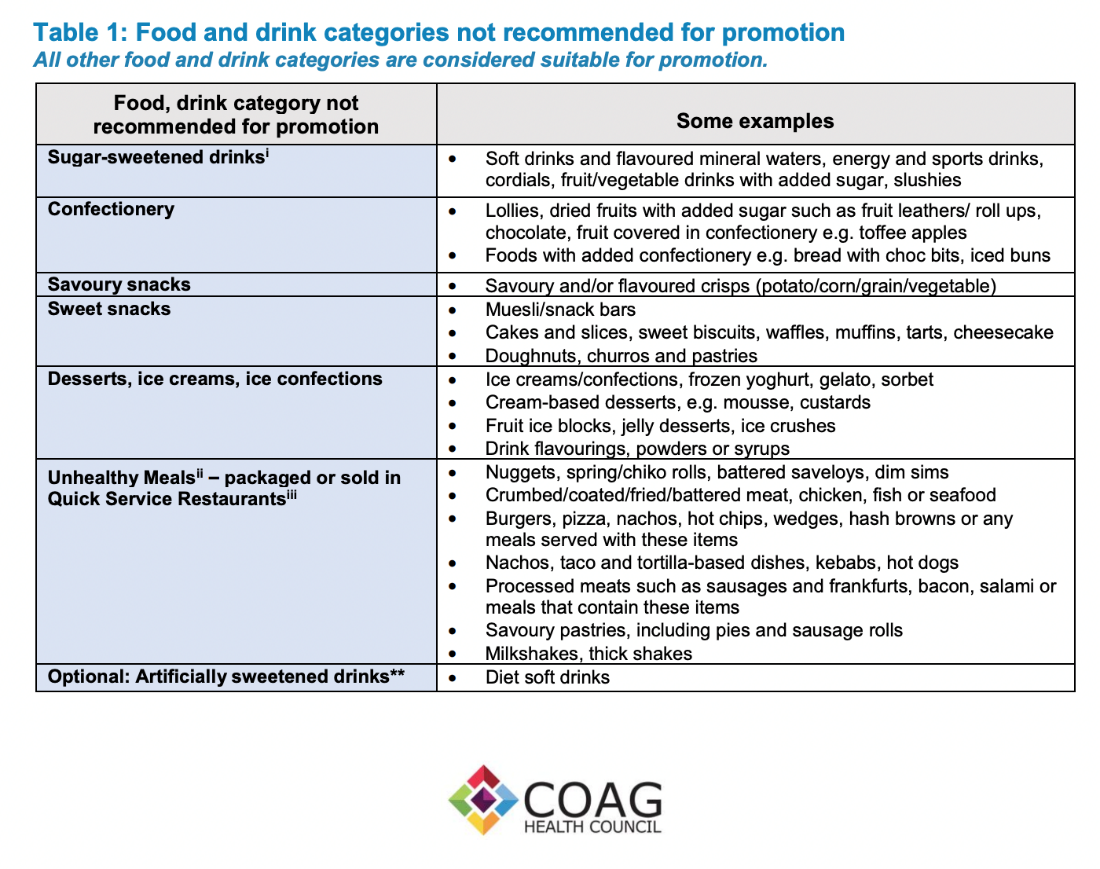


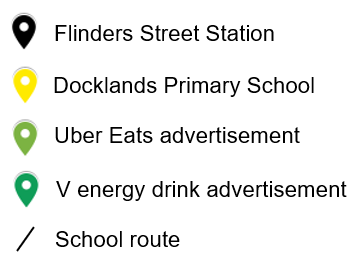

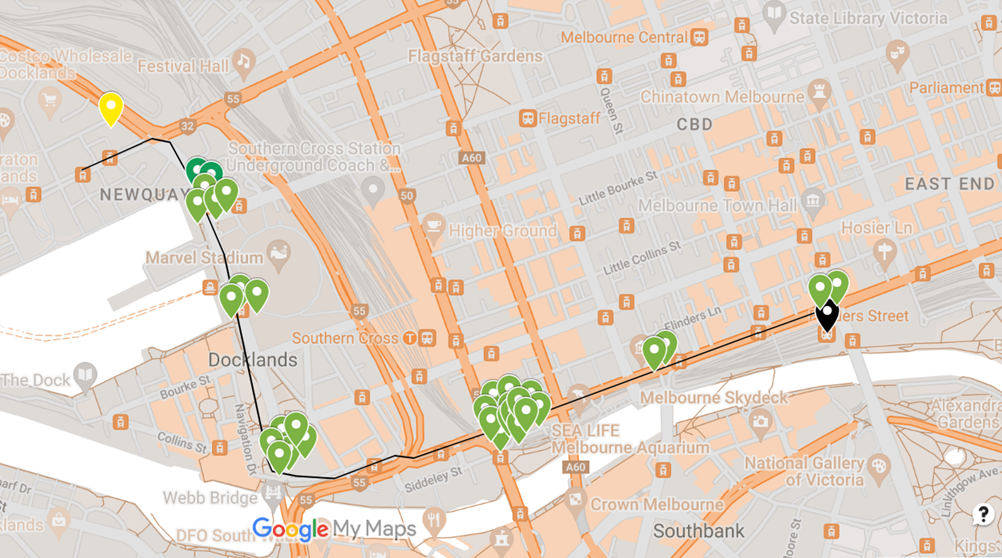


**Supplementary Figure 1. Density of Unhealthy ads on the Docklands Primary School route.**

**Supplementary Figure 2. Unhealthy advertisements, classified based on COAG**. The advertisements could contain one or more of the creative marketing strategies: promotional characters/ celebrity, premium offers/ competition, appeals to price, appeals to taste, appeals to emotion, reference to sport, relevant to local culture, reference to health, “new”/ “modern”, social corporate responsibility, and appeal to convenience
